# Assessing cell migration in hydrogels: An overview of relevant materials and methods

**DOI:** 10.1016/j.mtbio.2022.100537

**Published:** 2022-12-29

**Authors:** Anita Akbarzadeh Solbu, David Caballero, Spyridon Damigos, Subhas C. Kundu, Rui L. Reis, Øyvind Halaas, Aman S. Chahal, Berit L. Strand

**Affiliations:** a Department of Biotechnology and Food Sciences, NOBIPOL, NTNU- Norwegian University of Science and Technology, Trondheim, Norway; b Department of Clinical and Molecular Medicine, NTNU- Norwegian University of Science and Technology, Trondheim, Norway; c Department of Biotechnology and Nanomedicine, SINTEF Industry, Trondheim, Norway; d 3B's Research Group, I3Bs – Research Institute on Biomaterials, Biodegradables and Biomimetics, University of Minho, Headquarters of the European Institute of Excellence on Tissue Engineering and Regenerative Medicine, 4805-017, Barco, Guimarães, Portugal; e ICVS/3B's – PT Government Associate Laboratory, 4805-017, Braga/Guimarães, Portugal

**Keywords:** Hydrogel, Cell migration, Chemotaxis, Extracellular matrix, Three-dimensions, Scaffolds

## Abstract

Cell migration is essential in numerous living processes, including embryonic development, wound healing, immune responses, and cancer metastasis. From individual cells to collectively migrating epithelial sheets, the locomotion of cells is tightly regulated by multiple structural, chemical, and biological factors. However, the high complexity of this process limits the understanding of the influence of each factor. Recent advances in materials science, tissue engineering, and microtechnology have expanded the toolbox and allowed the development of biomimetic *in vitro* assays to investigate the mechanisms of cell migration. Particularly, three-dimensional (3D) hydrogels have demonstrated a superior ability to mimic the extracellular environment. They are therefore well suited to studying cell migration in a physiologically relevant and more straightforward manner than *in vivo* approaches*.* A myriad of synthetic and naturally derived hydrogels with heterogeneous characteristics and functional properties have been reported. The extensive portfolio of available hydrogels with different mechanical and biological properties can trigger distinct biological responses in cells affecting their locomotion dynamics in 3D. Herein, we describe the most relevant hydrogels and their associated physico-chemical characteristics typically employed to study cell migration, including established cell migration assays and tracking methods. We aim to give the reader insight into existing literature and practical details necessary for performing cell migration studies in 3D environments.

## Graphical abstract

**Figure:**
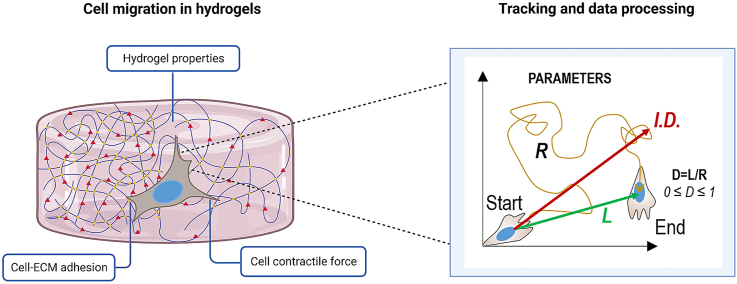

## 1 Introduction

Cell migration is a fundamental phenomenon in both physiological and pathological processes, such as in embryogenesis [1], where cells migrate to build the different organs and tissues in the body; in wound healing [2], where a collection of cells coordinates their motion to stabilise an injury; or in tumour progression [3], where cancerous cells invade the surrounding stroma toward the vasculature initiating metastasis. Other processes, such as bone remodeling, tissue regeneration, or immune response, also involve directed cell motility [4]. During the last decades, the mechanisms of cell locomotion have been a subject of intense research both *in vivo* and *in vitro*. The canonical view establishes that cell migration is first initiated by the adhesion of a cell (or group of cells) on the substrate forming focal adhesions [5]. Next, the cell polarizes in response to external stimuli reorganizing the inner actomyosin cytoskeleton to initiate migration. For this, the cell elongates membrane protrusions (typically, filopodia and lamellipodia) at the front edge and detaches the adhesions at its rear edge. This cycle is repeated in a highly coordinated and conserved manner, resulting into a migration path that can be either stochastic (random motion) or directional, depending on intrinsic and extrinsic factors [6,7]. These factors can be of different origins, including physical (e.g., the rigidity of the extracellular matrix – ECM), biochemical (e.g., the presence of chemoattractants), or a combination of both, which ultimately influences the motility of cells. Even though *in vitro* experiments have provided much insight into our understanding of how cells interact with and rely on their surroundings to acquire guidance for movement, cell locomotion is a more complex and less understood process *in vivo*. Mainly, the ECM is no longer considered a static physical support used by cells to adhere and hold together [8]. Instead, cells and the ECM co-exist in a synergistic relationship, where they physically and chemically interact. For instance, cells deposit proteins and reorganize the ECM altering its structural and biochemical properties [9]. Such cell-driven modification, in turn, alters the morphology and mechano-sensing mannerisms of the cell. Additionally, similar physical and chemical changes within the ECM are known to regulate the movement of cells in a directed and orderly manner [6,10]. Cells are inherently equipped with internal compasses that respond to physical and chemical gradients within their immediate microenvironment [11,12]. However, the exact molecular mechanisms that orchestrate these processes are not well understood and are an ongoing field of research.

Recent advancements in tissue engineering, microtechnology and materials science have permitted the study of three-dimensional (3D) cell migration with striking similarities to the *in vivo* scenario. In particular, biomimetic hydrogels have been widely employed as a biomaterial capable of reproducing the mechanochemical and biological properties of native tissue. Hydrogels can be engineered and precisely tuned in stiffness or biochemical moieties to allow investigation of the mechanisms underlying 3D cell migration in a highly controlled and reproducible manner. The field of hydrogels for cell migration studies is broad, with an extensive library of materials, fabrication methods, and availability of physical and chemical tailorability. Furthermore, advanced analytical techniques to monitor and characterize cell migration are available, with the need for automation and increased accuracy being a driving force.

This work provides an accessible overview of relevant biomaterials and methods for cell migration studies.We discuss the challenges of materials and techniques and address prospects of 3D cell migration studies in hydrogels. We focus on the hydrogels typically employed and discuss their main attributes together with relevant characterisation techniques. Finally, we discuss different imaging and analytical methods and resources available to monitor and characterize cell motility in 3D. Overall, this paper outlines relevant parameters to conduct 3D cell migration studies, and thus may serve as a practical experimental guide.

## 2 Implications of hydrogel properties on migrating cells

Hydrogels are composed of crosslinked hydrophilic polymers capable of taking up water resulting in swollen bulk materials with a high content of the aqueous solution, such as cell culture media or body fluids. Their significant liquid content, mechanical properties, and network permeability make them similar to the native tissue environment [13,14]. Therefore, engineered hydrogels can be employed as realistic *in vitro* ECM microenvironments for cell migration studies. Hydrogels are typically classified based on their polymer type, crosslinking mechanism, and responsiveness [15,16]. They are obtained from natural sources or can be synthesized, whereas natural polymers are often more complex and heterogeneous in chemical composition than synthetic ones. The polymerization process leading to hydrogel formation is based on chemical or physical crosslinking resulting in hydrogels with varying properties. For example, chemically-crosslinked hydrogels (through covalent bonds) result in more stable hydrogels over time than physically-crosslinked ones (e.g., through hydrogen bonding, ionic or van der Waals interactions, and molecular entanglements).

Meanwhile, physically-crosslinked hydrogels can form under milder conditions, e.g., changes in temperature, without the need to use toxic chemicals or harsh synthesis steps. This makes them suitable in studies where cells are incorporated before gelation. Finally, hydrogel properties originating from the polymer and crosslinking type can potentially be sensitive and respond differently to various external stimuli, such as pH, ionic strength, and temperature, among other factors. These characteristics can also be tailored to construct stimuli-responsive materials for specific applications, such as thermoreversible gels that can be produced at room or body temperatures [17].

3D cell migration depends on not only the intrinsic properties of the hydrogel, but also the cell type and the cells’ inherent capability to adapt according to the changes in the environment. In general, cells can exhibit different migration modes, namely mesenchymal and amoeboid motility, or a transitional state of migration, such as lobopodial migration [18]. In amoeboid migration, cells have rounded morphologies and form actin protrusions referred to as blebs [19,20]. In migrating cells, the nucleus is positioned in the middle of the cell body, with the centrosome, the centre connection of the microtubules, behind the nucleus pushing the cell forward. This amoeboid migration mode has a low to no dependence on matrix degradability and cell-matrix adhesion [21]. However, when cells migrate via lobopodial mechanisms, a hydrostatic pressure induces bleb formation, followed by the nucleus acting as a piston, resulting in forces exerted onto the ECM via tight adhesions [22]. Interestingly, lobopodial migration is adhesion-dependent but independent of matrix degradability. Hence, it is often considered an intermediate mechanism between amoeboid and mesenchymal migration. In mesenchymal migration, mature focal adhesions are formed mainly in the lamellipodia and filopodia for applying traction forces, with the centrosome typically positioned in front of the nucleus [21]. This is morphologically evident, where cells appear polarized in the direction of migration. In contrast to lobopodial migration, mesenchymal migration is highly dependent on matrix degradability and requires strong cell-ECM adhesions.

Hydrogel's physical and chemical nature can directly influence the extent, ability and manner in which cells migrate across these substrates. For example, cell attachment can be supported by adhesion ligands (e.g., RGD peptides) of the ECM. Additionally, translocation of the cell body can be affected by alterations in porosity, mechanical properties, and/or matrix degradability. Finally, hydrogel mechanical properties can also influence the ability of a cell to apply traction forces, consequently affecting the migration speed and/or the migration mode [23]. Herein, we focus on the effect of hydrogel network structure, mechanical properties, and grafting possibilities on 3D cell migration. However, it is essential to note that hydrogel characteristics are highly interdependent. Therefore, it is challenging to detangle properties and isolate one factor from the others.

### 2.1 Network structure

**Fig. 1:**
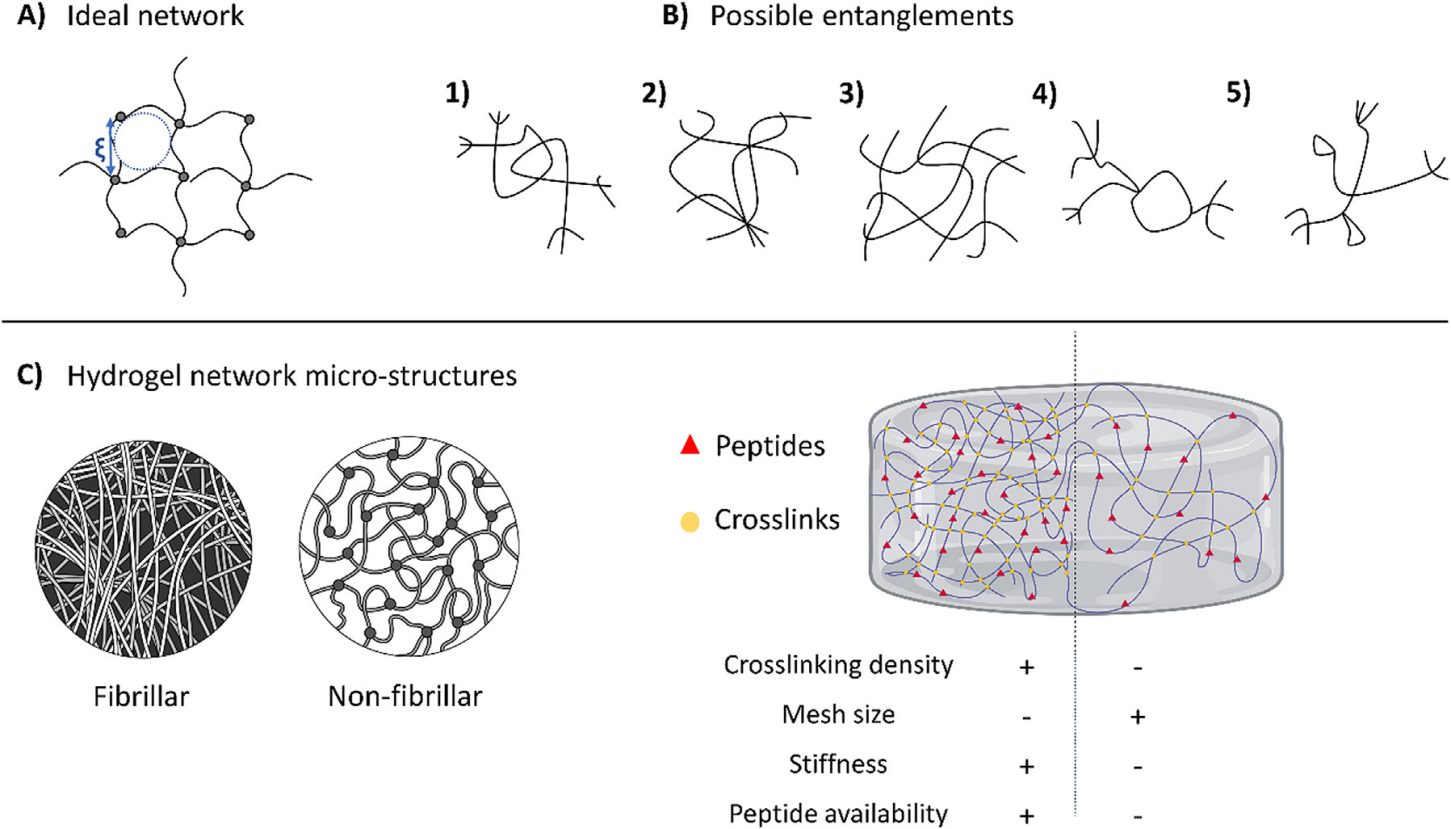
**Illustration of hydrogel networks. A)** Ideal hydrogel network showing the mesh size definition (ξ). **B)** Possible theoretical hydrogel entanglements: (1) tetrafunctional crosslinks, (2) presence of multifunctional junctions, (3) molecular entanglements, (4) presence of unreacted functionalities, and (5) presence of chain loops. **C)** Schematic of fibrillar and non-fibrillar *in situ* hydrogel network structures (left) and an illustration of a 3D hydrogel showing the interdependence of structural properties (right) [16,26].

The ECM can be viewed as a complex polymeric network with an interconnected 3D porous structure. The most crucial network parameters for 3D cell migration experiments are mesh size (ξ) and pore connectivity since these parameters can physically restrict or enable the passage of cells [24,25]. Hydrogel mesh size is the distance between two adjacent crosslinks. While the mesh size in an ideal hydrogel is well-defined (Fig. 1A), polymer strands can form other theoretical molecular entanglements and joints leading to distinct molecular networks (Fig. 1B). These possibilities lead to fibrillar (e.g., collagen and fibrin) or non-fibrillar (e.g., poly(ethyelene glycol) - PEG) network structures at the micro-scale (Fig. 1C) [16,26]. Therefore, a distribution of mesh sizes from uneven distribution of crosslinks is often presented. However, simplistic models of possible network structures offer a good representation (Fig. 1A and B). They can be used as the basis of calculations to estimate network structural characteristics, such as mesh size [27].

The optimal pore size of the hydrogel to enable cell migration depends on the biophysical properties of the ECM and cell type [27]. Mesh size across the polymer network is affected by crosslinking density, where higher crosslinking densities typically results in smaller mesh sizes. A smaller mesh potentially hinders cell migration, while a larger mesh size can translate to fewer adhesion sites and reduced mechanical support in 3D hydrogels (Fig. 1C). Hydrogels are either degradable or non-degradable by cells. For example, some natural hydrogels, such as collagen, Matrigel, and fibrin, can be proteolytically degraded by cell-secreted enzymes, such as matrix metalloproteinases (MMPs). Still, most synthetic hydrogels are non-degradable [28].

Nonetheless, some synthetic hydrogels can also be modified to become susceptible to degradation, e.g., by introducing protease sensitive crosslinking. Hydrogel degradability affects the range of pore sizes that could lead to cell migration. For example, Wolf et al. compared the speed of both MMP-dependent and MMP-independent migration of HT1080 sarcoma cells in a porous collagen hydrogel. They showed that in MMP-independent migration, where cells could not degrade the ECM, migration was more influenced by the pore size [29]. In degradable hydrogels with dense networks and small pore sizes, mesenchymal cells can migrate by deforming and degrading the matrix [30,31]. Other cell types, such as lymphocytes, dendritic cells, and tumour cells, can also employ alternative amoeboid migration modes to squeeze through the pores, including non-degradable hydrogels that are porous enough to permit their displacement physically [32]. In this regard, the porosity of non-degradable hydrogels to enable cell migration is limited to the cell nuclei size – the stiffest organelle of the cell – and its deformation ability [29]. The size of the cell nucleus is in the range of 3–15 ​μm, which is bigger than the pore size of many tissues [29,33]. However, native tissue contains interstitial spaces of pore sizes ranging between 0.1 and 30 ​μm in diameter; therefore, some cells need to squeeze through these pores to migrate [33].

Besides mesh size, fibre stiffness, thickness, and length have also been shown to affect cell spreading, attachment, and migration in fibrillar hydrogels [23,34]. For instance, Doyle et *al.* studied the migration of human forehead fibroblasts in four collagen hydrogels with various fibre thicknesses and porosities [23]. They showed that the cells made more protrusions and migrated faster on thicker fibres while aligning themselves along the fibre direction. Therefore, the alignment of hydrogel fibrils can also direct the motility of cells unidirectionally [35].

Different experimental methods are available to characterize the network structure of hydrogels (Table 1). These methods can be categorized as microscopy techniques for the direct measurement of the polymeric network or indirect methods to estimate the mesh size using theoretical models and scattering methods. The different microscopy methods cover length scales ranging from the micro-/nano-metric dimensions via atomic force microscopy and transmission electron microscopy to the mesoscopic scale via scanning electron and more conventional optical microscopy techniques. The latter provides a diverse toolbox, ranging from the most straightforward – but very limited – brightfield microscopy to more informative fluorescence-based methods, which can distinguish the different building blocks (e.g., materials, biological elements, etc.) of the cell-laden hydrogel. More sophisticated approaches are preferred depending on the composition or the characteristics of the hydrogel. Depending on the composition or the characteristics of the hydrogel, more sophisticated approaches are preferred. For instance, second-harmonic generation (SHG) microscopy is especially well-suited to characterize the endogenous components of the ECM, mainly collagen, in a very sensitive manner and without the need to stain the sample. An additional advantage of this method is its compatibility with standard confocal microscopy, which enables multiple ECM components and cells to be visualized together. However, confocal and SHG are limited in terms of their optical resolution and long acquisition times. New optical methods have emerged to characterize matrix architecture and composition to avoid this. Super-resolution and light sheet microscopy stand out due to their superior optical properties. Super-resolution microscopy overcomes the theoretical diffraction limit of light and improves the quality of images providing unprecedented details on hydrogel network elements. And light sheet microscopy solves the photobleaching/phototoxicity and long acquisition problems typically encountered by other optical methods when imaging large hydrogel samples.

**Table 1:** Direct and indirect techniques to characterize hydrogel network structures.

	Method	Applications	Limitations	Ref.
Microscopy/direct techniques	Atomic force microscopy (AFM)	High-resolution imaging of the hydrogel nano- and micro-topography in both native and dried conditions.	Limited to the surface of a hydrogel Difficult to use for soft hydrogels (G’ ​∼ ​few hundred Pa). Small image area.	[[37], [38], [39]]
	Transmission electron microscopy (TEM)	Powerful magnification of hydrogel elemental inner structure. Crystalline characterization.	Laborious sample preparation The sample needs to be prepared in thin slices.	[40]
	Scanning electron microscopy (SEM)	High-resolution imaging of hydrogel surface topography and information about its chemical composition using EDS detectors. 2D and 3D imaging of the hydrogel when combined with a focused ion beam.	Limited to dried samples. A harsh treatment is required to dry and coat the sample with a thin metal layer.	[[40], [41], [42], [43]]
	Optical Microscopy	*Brightfield* Affordable; Reduced phototoxicity; Simple to use	Low contrast; Poor resolution; Difficult to distinguish different cell types; mainly limited to hydrogel surface	[42,44,45]
		*Epifluorescence* Fast imaging of hydrogel network structure and content Dynamics of the hydrogel network	Photo bleaching Out-of-focus background. Photo toxicity	
		*Laser-scanning confocal microscopy* High-resolution imaging of hydrogel 3D network structure and dynamics	Photobleaching Time-consuming for large z-stacks.	
		*Second harmonic generation* Fast imaging of hydrogel structure in the native state Label-free imaging of collagen organization (+other proteins)	Restricted to a small number of structural proteins	
		*Super-resolution microscopy* Nanometric resolution of hydrogel network structure	Difficult to capture dynamic events	
		*Light sheet microscopy* Fast imaging of large hydrogel samples Reduced phototoxicity and photobleaching	Lower resolution due to beams scattering in deep samples	
**Indirect techniques**	Rheology	Gel elasticity determines crosslinking density Provides average mesh size by measuring elastic blob.	Limited to polymers exhibiting characteristics close to rubbers, well-described by Flory theory, or under small deformations in linear viscoelastic region	[36]
	Cryoporosimetry	Assumes water crystallizes in the polymeric network with a size related to polymeric mesh size distribution.	Inevitable overestimation upon water freezing due to possible network deformation.	[46]
	Low-field NMR	Characterizes a permanent dipole of the water filling the network and relates the protons' relaxation behaviour to the network mesh size distribution.	Purely a theoretical estimation.	[47]
	Release tests	Estimates average mesh size by measuring drug diffusion coefficient in the polymeric network.	It can be delicate and have a high error in mesh size estimation for low polymer concentration.	[48]
	Small-angle X-ray (SAXs)/neutron scattering (SANs)	Measures scattering of radiation from X-ray or neutron source on the sample	Only provides average values for structural parameters	[49]
	Dynamic light scattering (DLS)	To measure the diffusion of material within the hydrogel characterized by the correlation length of polymer chains in a crowded system.	Changes in polymer concentration can significantly affect the results	[50]

Indirect techniques use theoretical models to link experimentally measured parameters with mesh size based on certain assumptions. For example, rheology is based on rubber elasticity in Flory theory, in which crosslinks are considered as fixed points connecting four polymer chain ends. Thus, the measured shear modulus is linked to an average mesh size by the assumption of an ideal network (Fig. 1A) [36]. This works better for stiffer polymers or materials within the linear viscoelastic region under small deformations [16]. It is best to use more than one method and compare the results to find the best indirect way to get the hydrogel mesh size. Since each approach is based on its own model describing the network (Fig. 1), it is essential to choose a method closer to the structural architecture of the actual network of a specific hydrogel.

### 2.2 Mechanical properties

Human tissues display a broad range of stiffness from ∼20 ​Pa of adipose tissue to ​∼ ​GPa of bone [51]. The constant interaction between cells and the ECM causes a continuous restructuring of the cellular environment in which a perturbation in matrix stiffness may alter cells’ morphology, phenotype, and migration capacities [52,53]. Indeed, a recent study showed that stiff environments, such as in epidermis tissue, can affect the intracellular dynamics of T-cells and, therefore, their protruding capacity, influencing their motility patterns [54].

**Fig. 2:**
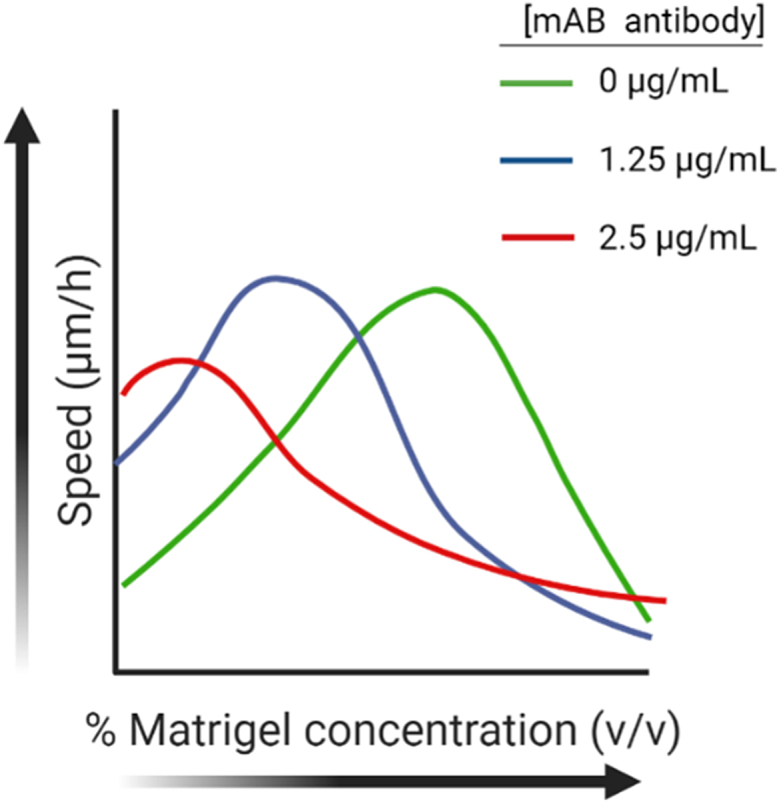
Biphasic relationship between cell migration speed and Matrigel concentration, corresponding to stiffness. Increasing ligand inhibitor (mAB antibody) shifts the maximum migration speed towards lower Matrigel concentration (softer matrices) [55].

Cell migration depends on mechanical matrix properties as an interplay between cells’ inherent contractility and ECM stiffness, affecting cell adhesion properties, such as the maturation, stabilization, lifetime, size, and disassembly of focal adhesions [23,55]. For example, human foreskin fibroblasts were shown to migrate faster inside stiffer collagen gels. However, by reducing the cell contractility and adhesion stability, cells migrated faster in softer gels while slowed down in stiffer ones [23]. Another study combining simulations and experiments showed that human prostate carcinoma cells (DU 145) migrated in Matrigel, exhibiting a biphasic relationship between migration speed and matrix stiffness with the highest speed at an intermediate Matrigel concentration (Fig. 2) [55]: Increasing Matrigel concentration by two folds were shown to duplicate ligand density and enhance stiffness by five folds. Increasing the concentration from 50% to 65–70% resulted in a higher migration speed of the DU-145 ​cells. However, a further increase in concentration reduced the migration speed due to the increment in ligand density. Introducing ligand inhibitors led to cell migration becoming less and less dependent on ligand density. Therefore, the maximum speed of migration shifted towards softer matrices. Next, increasing fibronectin content in the Matrigel hydrogel reduced the migration speed of the DU-145 ​cells in the same study [55]. β1 integrin blocking antibody was added to inhibit cell binding to the matrix to manipulate cell-matrix adhesiveness. In the presence of this antibody, the migration speed displayed a biphasic behavior with a maximum value shifting towards higher fibronectin concentrations as the binding to integrin was progressively inhibited. Nevertheless, the addition of fibronectin did not change Matrigel stiffness significantly.

Studies using less complex hydrogels, such as PEG and alginate, showed a more straightforward relationship between cell migration and stiffness. Increasing hydrogel stiffness has been reported to hinder cell migration in PEG, alginate conjugated with Matrigel, and RGD-alginate hydrogels [32,56,57]. For example, mouse pre-osteoblastic cells (MC3T3-E1) display limited motility in soft, MMP non-degradable PEG hydrogels, while increasing stiffness inhibited migration in these hydrogels altogether [32]. However, a stiffness increase in MMP-degradable hydrogels did not hinder migration entirely but reduced cell speed [32].

Natural tissue ECMs and most biological materials display complex mechanical properties, exhibiting time-dependent properties including viscoelasticity, viscoplasticity, non-linear elasticity and heterogenous behaviour depending on their location within the body [58,59]. The ECM can affect cell migration both regarding time and force scales of cell-ECM interactions where cells perceive the environment through their membrane and respond by reorganizing cytoskeletal elements. Hydrogels can be engineered to mimic the mechanical behaviour of the ECM, particularly viscoelasticity. Viscoelastic material presents behaviour between elastic solids that store energy (storage modulus) and viscous liquids, capable of dissipating energy (loss modulus). Stress-strain measurements are performed under stress- or strain-controlled conditions to distinguish between the elastic and viscous components of the viscoelastic materials. Measuring stress change over time under a specific strain provides hydrogel relaxation, while measuring deformation changes with time under a certain stress gives creep compliance. Stress relaxation in 3D cell migration is vital because as cells move through a hydrogel-based ECM, traction forces are applied to the polymer network. The hydrogel may react with force or dissipate the energy [60,61]. Some examples of hydrogels where stress relaxation properties can be tuned are RGD-alginate by changing RGD content and hyaluronic acid (HA) combined with collagen [[62], [63], [64], [65]]. A range of stress and strain assays relevant to cell-ECM interactions are probed using a rheometer to measure the stress relaxation of the hydrogel. Then, the strain is held constant while the load is recorded as a function of time [64]. Due to the time-dependent mechanical properties of hydrogels, measuring their mechanical properties can be divided into macro- and micro-scale methods in time or frequency domains. On a macro scale, a rheometer can be used for static (stress relaxation test, creep) or dynamic (frequency-dependent rheology, cyclic loading) mechanical tests [64,66,67]. Alternatively, on a micro-scale, viscoelasticity can be measured by indentation methods, such as depth sensing, scanning probe microscopy-based methods (e.g., atomic force microscopy – AFM) [68], or particle-based micro-rheology (passive or active) [[68], [69], [70]]. Passive particle-based micro-rheology can measure the interior of the gel and is useful for softer hydrogels [70]. Conversely, active particle-based micro-rheology is used for stiffer gels [71]. Microscale measurements are more relevant to the interaction scale of cells with materials.

### 2.3 Incorporation of peptides

Incorporating peptides is an essential step in synthetic ECM engineering, which helps design environments with moieties more similar to natural tissues. Typically, cell adhesion peptides have been either covalently or ionically anchored to hydrogels that lack bioactivity in their unmodified forms, such as alginate, agarose, and PEG [72]. This allows for a systematic investigation of cell receptors and ECM interactions, which in turn affects cell migration. The synthetic peptides RGD, IKVAV, and YIGSR have been massively employed due to their efficiency in promoting cell adhesion. Nevertheless, other peptides derived from collagen, laminin, fibronectin, vitronectin or elastin have also been utilized. Undoubtedly, RGD-based short amino acids are the most used peptide in tissue engineering. For example, varying RGD content in PEG hydrogels affected the morphodynamics of hMSCs (velocity, persistence length) and the number of migrating cells [32]. Incorporating single peptides or a combination of them can be challenging but it could potentially improve our understanding of specific cell-ECM interactions and help develop new strategies to control cell migration. Essential factors to consider when selecting peptides are solubility, concentration, stability, and the binding method used to link the peptide to the polymer substrate [72]. For a more in-depth discussion on the selection of peptides for cell migration, we recommend referring to Huettner et al. [72].

As a cell adheres to a peptide-functionalised hydrogel, traction forces are applied by the cell, initiating an adhesion-mediated migratory process (mesenchymal migration). However, the lack of anchoring points does not directly render a cell incapable of migrating. This is because some cells can migrate via an adhesion-independent mechanism, referred to as blebbing. Indeed, some cells, such as cancer cells, can switch from one mode to another to maximize the efficiency of motion [27]. Furthermore, the selection of migratory ways does not solely depend on the presence or absence of peptides, but also on the inherent mobile characteristics of the cell type itself. In addition, environmental features can limit the magnitude of cell-substrate adhesion, the extent of physical confinement, and the capacity for cell contractility [73]. Therefore, efficient cell migration is ultimately the result of the interplay of interactions and contributing forces and can collectively influence cells to adopt a spectrum of migratory modes ranging from mesenchymal to amoeboid and often somewhere in between.

### 2.4 Hydrogels as customizable substrates

Hydrogels of natural polymers have been used in cell culture for many years and can provide more physiologically-relevant environments than traditional 2D cultures. However, they can be variable in quality and complex in composition, leading to batch-to-batch variations. On the other hand, hydrogels of synthetic polymers are often more uniform in their composition, but they may not provide the same level of mimicry as the natural ECM derivations. While some natural hydrogels can support various cell functions, synthetic hydrogels provide flexibility towards chemical reactions and opportunities to isolate factors influencing cell migration for bottom-up approach studies. In this section, we concentrate on the hydrogel polymer material and discuss methods to modulate hydrogel properties with a focus on collagen, gelatin, Matrigel, alginate, and PEG hydrogels.

#### 2.4.1 Collagen

Collagen hydrogels can be formed physically by changing temperature and can be thermally reversible. Collagen can also be chemically crosslinked by covalent crosslinking (e.g., glutaraldehyde) [74]. In humans, 28 different types of collagens have been described as playing an essential structural role in most tissues [75]. Among them, collagen I is the most abundant collagen in the human body. It has a length of 300 ​nm and forms 67 ​nm banded fibrils. At low concentrations, collagen fibrils tend to entangle into thick fibers, due to the limited amount of nucleation sites, whereas, at high concentrations, they tend to form more rigid nematic-like structures [76]. Several factors have been used to modulate collagen I properties, ranging from collagen source, the extraction process, concentration, pH, temperature, ionic strength, and coatings (e.g., fibronectin and laminin) [29,74,[77], [78], [79]]. Collagens have different crosslinking degrees depending on their source [29]. The collagens extracted from bovine or human dermis have a higher degree of crosslinking in comparison to the low crosslinking degree in rat and mouse tails. Highly crosslinked collagens can be treated with pepsin to remove most of the telopeptide sites. This treatment has shown to result in collagens assembling with delay and form larger pore sizes and longer fibrils compared to non-treated collagen [29,77,80]. Also, it has been shown that variations in collagen concentrations have a direct influence on collagen pore size [77].

Changing pH and temperature affects polymerization rate, fibril thickness, network density, and, ultimately, the mechanical properties of collagen [74,78]. Collagen hydrogels made at 37 ​°C and neutral pH show a homogenous and highly reticular mesh. Reducing the temperature or pH increased the pore size and produced thicker fibrils due to enhanced of fiber self-assembly at a lower temperature, and therefore, resulting in a more heterogeneous matrix. A matrix with thicker fibrils also produces stiffer gels [74,78]. Ionic strength also affected the rate of fibril formation in collagen gels; reducing pH and increasing ionic strength delayed the rate of fibril formation [79]. Changing ionic strength also affects the collagen microstructure, in which increasing ionic strength results in the formation of more packed fibrils, while loosely packed fibrils are formed under lower ionic strength [79]. Increasing ionic strength also led to a finer substructure and sheet-like appearance [79]. To control the orientation of collagen fibrils, magnetic field [81], electrochemical fabrication [82], stretching techniques [83], and bioprinting [84] have been used [74].

#### 2.4.2 Gelatin

Gelatin is the proteinaceous substance derived from collagen by physical, chemical, or enzymatic hydrolysis, breaking collagen's triple-helix structure into single-stranded molecules. Therefore, it exhibits similar chemical and biological properties to collagen but lacks the fibrillar structure. Like collagen, gelatin-based hydrogels also contain RGD motifs and MMP cleavable peptides. To produce gelatin, acidic or alkaline treatments are employed, resulting in two types of gelatin, A and B, exhibiting net positive and net negative charges, respectively, enabling further modifications and applications. Gelatin hydrogels can form by cooling or enzymatic and chemical crosslinking [52,85]. The thermal hydrogels lack mechanical stability and form weak gels [13,52]. Hence, gelatins are commonly used in a chemically-modified form, such as gelatin with, e.g., methacrylate (Gel-MA) to create covalently crosslinked hydrogels with tunable stiffness or blended with other polysaccharides, such as alginate or chitosan, to improve the mechanical properties [[85], [86], [87]]. The source and extraction process of gelatin, like collagen, determines its molecular weight and amino acid proportions, which again influence its mechanical properties [88].

#### 2.4.3 Matrigel

Matrigel is a mixture of glycoproteins and small molecules extracted from the basement membrane of the Engelbreth-Holm-Swarm (EHS) mouse sarcoma tumor. It contains approximately 60% laminin, 30% collagen IV, and 8% entactin [89]. Matrigel also contains proteoglycans (e.g., heparan sulfate), cytokines, and growth factors, such as EGF, ODGF, and other elements from the EHS cell line [52,90,91]. It is commercially available in a frozen form and gels upon heating. Matrigel contains entactin or heparin-binding proteins that can interact with laminin and collagen IV to self-assemble into a gel, for example, by providing nucleation sites for fibril formation. When mixed with Matrigel, this interaction can lead to changes in the microstructure of collagen hydrogels when mixed with Matrigel, resulting in wider fibrils and larger pores [74,92]. The gelation of Matrigel may occur as fast as ∼30 ​min at 37 ​°C and is thermally reversible. Matrigel is inherently inconsistent in its molecular composition and shows batch-to-batch variability, which difficult the mechano-chemical modulation of the material [93]. Changing Matrigel concentration, the addition of fibronectin, and β1 integrin blocking antibody have been used to influence 3D cell migration in Matrigel [55].

The gelation of Matrigel may occur as fast as ∼30 ​min at 37 ​°C and is thermally reversible. Matrigel is inherently inconsistent in its molecular composition and shows batch-to-batch variability, which difficults the mechano-chemical modulation of the material [93]. Changing Matrigel concentration, the addition of fibronectin, and β1 integrin blocking antibody have been used to influence 3D cell migration in Matrigel [55].

#### 2.4.4 Alginate

Alginates are natural linear polysaccharides containing various sequences of the two monomers β-**d**-mannuronic acid (M) and α-**l**-guluronic acid (G) [94]. Alginate hydrogels are commonly formed via crosslinking with multivalent cations, such as Ca^2+^, but can also be covalently crosslinked by, e.g., peptides in amidation reactions [95]. Consecutive G (G-blocks) are mainly responsible for alginate gelation in ionically-crosslinked hydrogels commonly used in tissue engineering [96]. The gelation and properties of alginate hydrogels can be affected by alginate composition (G-block content and length), alginate concentration, molecular weight, type and concentration of crosslinking ions [37,[97], [98], [99]]. Alginate can be extracted from seaweed or bacteria, such as *Azotobacter vinelandii* [100]. Increasing G content and alginate concentration increases crosslinking density and hence stiffness. Higher alginate concentration also leads to gels with smaller pores [37]. Commercially-available alginates have molecular weights between 32,000 and 400,000 ​g/mol [97]. High molecular weight alginate displays high viscosity, which can be undesirable in handling, but is beneficial in forming stiff and stable hydrogels [98]. Interestingly, alginate molecular weight has been used to modulate hydrogel viscoelasticity and stress-relaxation properties. Varying molecular weight and alginate concentration simultaneously allow for independently controlling viscoelasticity and gel stiffness [62,64,101].

Divalent ions, such as calcium, barium, and strontium, are typically used for alginate gelation. It is shown that strontium and barium can crosslink with shorter G-blocks and form stronger crosslinks than calcium, but calcium can also crosslink blocks of alternating M and G (MG-blocks) [96,102,103]. Calcium is commonly chosen amongst the divalent ions to make alginate hydrogels for tissue engineering. The reaction occurs either by internal gelation using slowly hydrolyzing calcium salts, such as CaCO_3_ and glucono-δ-lactone or by external gelation using highly soluble calcium chloride [104]. Calcium concentration is shown to affect hydrogel stiffness and porosity. Increasing calcium concentration leads to gels with higher stiffness [98]. A slight increase in calcium concentrations (5 ​mM) in 1.5% (w/v) alginate hydrogel did not affect the pore size. However, larger increase from 36 to 144 ​mM Ca^2+^ has depicted a decrease in pore size from 247.5 to 30 ​μm [37,105]. Finally, a higher calcium concentration of calcium has even led to the stacking of G-blocks resulting in larger pore sizes [106].

Cell culture media content can also affect alginate hydrogels. For example, phosphate can interact with the Ca^2+^ in the alginate hydrogels and act as a chelator and monovalent sodium ions can exchange the crosslinking ions and destabilize the gel. Alginate hydrogels are known to be non-toxic and inert towards cells. Therefore, they have been used for 3D cell migration studies in peptide-grafted forms or mixed with other hydrogels, such as Matrigel and collagen [56,107]. For example, MMP-degradable alginate can be made by crosslinking with protease-degradable peptides, such as PVGLIG, and peptides necessary for cell attachment, such as RGD peptides, can be coupled to alginate [57,[108], [109], [110]].

#### 2.4.5 PEG

Poly(ethylene glycol) is a well-defined, synthetic, hydrophilic polymer with low polydispersity synthesized by the polymerization of ethylene oxide. PEG composite macromers can be made from diverse starting materials with various end groups, such as alcohol, acrylate, methacrylate, allyl ether, maleimide, vinylsulfone, methyl ether, amine, *N*-hydroxysuccinimidyl ester (NHS), and vinyl ether groups allowing flexibility in chemical modification and crosslinking. PEG hydrogels are shown to be bio-inert and maintain cell viability, they are chemically well-defined, and multiple chemistries can be used for their formation and modification, including the formation and removal of crosslinks by light [111].

Typically made by covalent crosslinks, PEG hydrogels have the advantage of forming stable hydrogels that allow for high tunability over hydrogel properties [112]. The mechanisms of fabricating covalently crosslinked PEG hydrogels are chain growth polymerization (e.g., photopolymerization), step-growth polymerization (e.g., Michael-type addition, click chemistry), or a combination of both [112,113]. Chain growth polymerization requires an active center (e.g., a radical) to attack a monomer. In contrast, step-growth polymerization involves two multifunctional monomers with functionality >2 to be mutually reactive towards each other and interact stoichiometrically. Chain growth polymerization also has the advantage of occurring within minutes avoiding exposure to heat or factors affecting cell encapsulation. However, it can lead to network non-idealities. Step growth polymerization has fewer network non-idealities during gelation, allowing accurate mathematical predictions of the reaction and high crosslinking density control [112]. Depending on their end groups, PEG macromers can crosslink to form hydrogels with crosslinking chemistry. For example, vinyl end groups can be reactive with a radical initiator. Radical initiators can be activated chemically by redox reactions or with light. Acrylate and methacrylate end groups can crosslink in the chain and step-growth polymerizations. Other groups, such as vinyl sulfone, maleimide, vinyl ether and allyl, can undergo step growth network formation.

PEG hydrogels can also be crosslinked with MMP-cleavable peptide sequences and adhesion ligands, such as RGD, to build bioactivity on their bio-inert background. Modulating PEG hydrogel properties depends on the method chosen for hydrogel fabrication. In general, parameters such as increasing polymer concentration and crosslinker, lead to increasing crosslinking density. Ehrbar et al., covalently crosslinked PEG hydrogels with peptides by using the enzyme transglutaminase factor XIII to connect glutamine acceptor substrate and lysine donor substrate to form MMP degradable peptides [32]. Depending on hydrogel degradability and stiffness, matrix stiffness was changed by varying polymer concentration and showed 3D migration of mouse preosteoblastic cells (MC3T3-E1). While increasing crosslinking density limited and further inhibited cell migration, the MC3T3-E1 cells in soft non-degradable PEG hydrogels migrated to a similar degree as in the soft degradable hydrogels supporting both proteolytic remodeling migration and MMP-insensitive migration mode [32].

## 3 Cell migration

### 3.1 Mechanisms of directed cell migration

Directed cell migration is critical for numerous physiological, pathological, and developmental events where cells move directionally either as individual entities or collectively, such as in cancer invasion or embryonic development [114,115]. Individual motile cells can also display random trajectories moving in a Brownian-like manner with no preferential direction. This random motion can be rectified by adding an external stimulus of mechanical or (bio-) chemical origin to attract cells [[116], [117], [118]]. In both cases, the migration of cells can be either gradient-dependent or gradient-free, enabling a rich and complex portfolio of migration mechanisms. In the following, we describe the main types of cell migration mechanisms and the typical experimental strategies employed for their investigation.

#### 3.1.1 Mechanical-based

Mechanical-based cell migration mechanisms include *durotaxis, topotaxis,* or *curvotaxis*. In *durotaxis,* cells follow gradients of extracellular mechanical stiffness typically migrating from soft to rigid regions (positive *durotaxis*). Reverse or negative, *durotaxis* where cells migrate from rigid to softer regions has also been observed [119]. In conventional *durotaxis* experiments, photosensitive hydrogel surfaces are manufactured with increasing levels of crosslinking and rigidity. For this, dynamic UV-irradiation is usually employed where an opaque mask moves at a constant speed on top of the hydrogel during irradiation, causing increasing modifications of the physicochemical properties of the hydrogel network. This modification can be regulated by varying the sliding speed of the mask resulting into different rigidity gradient slopes [120]. *Durotaxis* has been well documented in different cell types *in vitro*, even though its molecular basis is still inadequately understood and it is *in vivo* relevance still needs to be determined [121]. Traditionally, *durotaxis* has been studied on planar surfaces and single cells. However, it has also been reported in multi-cellular clusters of epithelial cells and, interestingly, in 3D spheroids, showing the potential of *durotaxis* to operate in native-like scenarios [122]. Indeed, durotactic responses have been observed using complex *ex vivo* systems with *in vivo* relevant stiffness [123,124]. Therein, cells migrated directionally, suggesting that this mechanism may also occur *in vivo.*

Cells can also migrate along gradients of topographical features in a mechanism termed *topotaxis* [125]. This phenomenon is cell-dependent, meaning that cells can migrate either in one direction or the opposite along the gradient depending on their transcriptomic status or as a result of simple scaling arguments. Conventional *topotaxis* assays involve the seeding of cells in 2D surfaces containing a topographic gradient. However, *topotaxis* can also be observed in 3D, with cells being encapsulated within a hydrogel environment with local topographic features distributed in a spatially graded fashion. Note that this increase in density may also trigger a local rise in rigidity. Therefore, in specific scenarios, it is challenging to distinguish whether directed cell migration results from *topotaxis*, *durotaxis*, or a combination of both. In this regard, additional experiments might be necessary to disentangle both effects.

Next, in *curvotaxis*, cells respond to small changes in curvature variations to undergo directed locomotion [126]. In *curvotaxis*, cells prefer to locate in concave regions avoiding convex ones, which is determined by a tight interplay between the cell nucleus, cell adhesions, and the cytoskeleton. Like the former migration mechanisms, *curvotaxis* has been mainly observed *in vitro* using static 2D sinusoidal-like surfaces. That is, this type of cue does not completely surround cells. However, the high complexity of the *in vivo* scenario may enable the directed migration of cells through *curvotaxis*, particularly during embryonic development, where cells are exposed to continuous topographic changes, particularly in curvature, due to tissue growth. Other mechanical-based methods used to bias cell migration include *electrotaxis* (changes in electric field) [127] or *barotaxis* (changes in hydraulic pressure) [128]. Despite not being the preferred option, these methods have been demonstrated to be well-suited particularly when combined with cell-laden hydrogels and microfluidics to promote directional cell migration. One of the main advantages of these methods is the possibility to control the activation of the signal by, e.g., switching on-off the electric field or balancing the hydraulic resistance and dynamically controlling the intensity of the cue and, therefore, the slope of the physical gradient. Finally, other mechanical-based methods employed to guide the motion of cells include *contact guidance* or *ratchetaxis.* In typical *contact guidance* experiments, cells move in response to anisotropic topographical features, such as physical grooves. For *ratchetaxis*, a periodic array of asymmetric topographical features is employed to physically impose the polarity of cells to induce their directional motion [129]. The rational of using a periodic array is to maintain the memory of migration and prevent cells from depolarizing and reverse their motion. Note though that these two strategies also fit within the (bio-) chemical category since cells may behave similarly using micropatterned adhesive lines or asymmetric features. Therefore, they may be considered as hybrid mechanisms.

#### 3.1.2 (Bio-) chemical-based

Many pathophysiological processes involving directed cell migration are a consequence of *chemotaxis* or *haptotaxis*, where cells respond to gradients of soluble or surface-anchored factors, respectively, and migrate toward the direction of increasing concentrations of the chemoattractant (*e.g.,* growth factors, peptides, metabolites, or chemokines) [130,131]. For example, gradients of growth factors (*e.g.,* VEGF) have been shown to be involved in the directed motion of cancer cells toward the microvasculature initiating metastasis [132] or during angiogenesis [133]. Other examples include the migration of immune cells towards an external insult (*e.g.,* infection) or the directed migration of fibroblasts and epithelial cells during wound healing to repair the damaged area and close the gap (*e.g.,* inflammatory cytokines gradient). Due to its simplicity and physiological relevance, chemotaxis is the most utilized method for investigating directed cell migration *in vitro*. In 2D *chemotaxis* experiments, two interconnected containers are typically employed, one containing the chemotactic agent for generating a gradient by diffusion. Similarly, in 3D *chemotaxis*, cells are usually embedded within a 3D hydrogel located in between the two compartments with a high and low concentration of chemoattractants that diffuse, generating the gradient. A limiting factor of this strategy is the difficulty of producing stable gradients that do not change over time. To solve this, small chemokine-containing capsules encapsulated within the hydrogel have been developed to release well-controlled quantities of the compound with a precise control on their degradation rate and, therefore, on gradient stability [134]. This method can generate local gradients of a chemokine, which can interact with cells. Interestingly, these capsules can also be actuated externally to promote the release of the compound [135].

A myriad of alternative gradient generation strategies has been employed to generate gradients for cell migration studies. Undoubtedly, Transwell systems are preferred due to their efficacy and simplicity. In this type of assay, the bottom compartment is filled with a chemoattractant that diffuses toward the upper reservoir attracting the cells typically located within a hydrogel [136]. This method is compatible with moderate high-throughput, thus enabling the parallelization of experiments. However, one of its main limitations is the difficulty of imaging cell migration in real-time. To circumvent this, 3D hydrogels can be directly soaked into a chemoattractant solution to gradually generate a gradient by diffusion. This immersion-based approach can generate large-scale soluble or surface gradients depending on the material's affinity of the material with the chemoattractant. Despite being one of the most straightforward procedures to generate biochemical gradients, the limited control on gradient slope threatens its physiological relevance [137]. Microfluidics has demonstrated a superior capability to create gradients with well-controlled lengths and slopes by exploiting the unique features of manipulating fluids within micro-sized channels. Under these conditions, viscous forces dominate over the inertial ones and fluid shows a laminar flow, that is, low *Reynolds* numbers. As a result, two (or more) fluids flow along a microchannel mix mainly by diffusion across their interface. Therefore, a few centimeters of microchannel lengths of few centimetres are needed to increase the interfacial contact between two fluids to completely mix. This particular effect can generate well-controlled gradients of chemokines within microfluidic systems to promote directed cell migration. For this, Y-shaped microfluidic systems encapsulating cell-laden hydrogels are mainly utilized for *chemotactic* and/or *haptotatic* cell migration studies. Despite the high control on gradient slope, this can slightly change along the channel due to diffusion. To solve this, cascade-based microfluidic designs, where the flow of each channel splits into two, can provide well-defined and highly stable concentration profiles, which can be theoretically predicted by knowing the initial concentrations of the injected compounds, chip architecture, and flow rates [138]. In all these cases, the microfluidic chip can be embedded with a 3D cell-laden hydrogel.

The above-mentioned techniques can also be used to generate gradients of reactive groups to tether a chemotactic compound via covalent and ionic bonds or complex formation. This allows the modification of the backbone of polymers within the hydrogel, enabling control of the presentation and release kinetic of chemotactic compounds [139]. A chemokine's release kinetics and presentation expression determine its efficacy and whether its effects are short or long-lived. On the one hand, some chemokines impose their effects when provided in bulk as a burst release to cells, while others have proven more effective in attracting cells when released over a long period in a controlled manner. For example, stromal-derived factor-1 alpha (SDF-1a) is a small chemokine belonging to the CXC subfamily of chemokines and is known for its potency in recruiting stem cells [140]. Its effects are most efficient when released in a gradual and long-lasting manner. As a result, various strategies have been devised that comply with the hydrogel loading capacity while enhancing the chemoattractive effect of SDF-1a on stem cells [139]. The selection of a chemotactic factor to induce cell migration depends on the target cell type. However, many cell types are known to respond to either CC, CXC, CXC3, or XC subfamilies of chemokines, as outlined in Table 2. The main factor that delineates these chemokines into subfamilies is related to the location of cysteine residues in relation to the *N*-terminus [141]. There are undoubtedly a wide range of chemokines that can stimulate the migration of specific cell types relevant to fields, such as cancer, immunology, wound healing and regenerative medicine (Table 2) [142,143].

**Table 2:** Main chemokines used to stimulate the migration of cells for applications in tissue engineering, regenerative medicine, wound healing and cancer biology.

Cell type	Chemokines	Relevant applications	Ref.
hMSCs	SDF-1a CCL3/5/15 CXC10 PDGF AA/BB	Tissue engineering and regenerative medicine	[144] [145] [146] [147] [148]
Fibroblasts	CCL5/15/20/22/25/27/28 CXCL1/11/13 CXC3CL1, XCL1	Tissue-specific model systems, wound healing	[149] [146]
Endothelial cells	VEGF	Angiogenesis	[150] [151]
Immune cells (T cells, NK cells, macrophages, neutrophils, mast cells and dendritic cells)	CXCR3/4 CXCL 9/10/11/12 CCL 2/4/5/6 CCR2/4/5/6	Cancer biology and immunology	[152] [153] [154]

In addition to the small chemokines and recombinant growth factors mentioned in Table 2, naturally available growth factors have been sought after for use in regenerative medicine to attract a variety of reparative cells [155]. Platelet lysates (PL) and platelet-rich plasma (PRP) derived from the blood have recently gained popularity for being an abundant and easily accessible source for growth factors [156]. Many biomaterials have incorporated PL and PRP due to their availability and for providing physiologically relevant concentrations of pro-regenerative and pro-inflammatory mediators [148,[156], [157], [158]]. Many biomaterials have incorporated PL and PRP due to their availability and for providing physiologically relevant concentrations of pro-regenerative and pro-inflammatory mediators [148,157,158]. While blood derivatives harnesses the synergistic effects of multiple growth factors and chemokines, essential factors need to be considered before its use in hydrogels. For instance, the polymer backbone and cross-linking mechanism must not physically or chemically impede the release of growth-factors permanently, preventing a chemotactic gradient from forming. In these cases, inert hydrogels, such as PEG, can serve as reservoirs for growth factors to limit the possibility of interaction with the crosslinked polymer network [158]. Additionally, blood derivatives present significant batch-to-batch variation and often require the pooling of samples. Next, an anti-coagulant, such as citrate or heparin, may be used to prevent growth factors from being precipitated and enhance availability for surrounding cells. Ultimately, striking a delicate balance between the release and retention of numerous growth factors from a single hydrogel construct over time can be challenging to execute carefully. However, evidence suggests that using multiple growth factors simultaneously can indeed be beneficial in eliciting a higher degree of cellular response [159].

### 3.2 Experimental methods to study cell migration using hydrogels

Recent advances in nanotechnology and microfabrication tools have resulted in various micro-engineered devices that can integrate 3D hydrogels to investigate different aspects of cell migration. These devices differ in their designs (simple *vs* complex), modalities (static *vs* dynamic), versatility (specific vs multi-functional), or fabrication material (soft elastomer *vs* solid polymer).

**Fig. 3:**
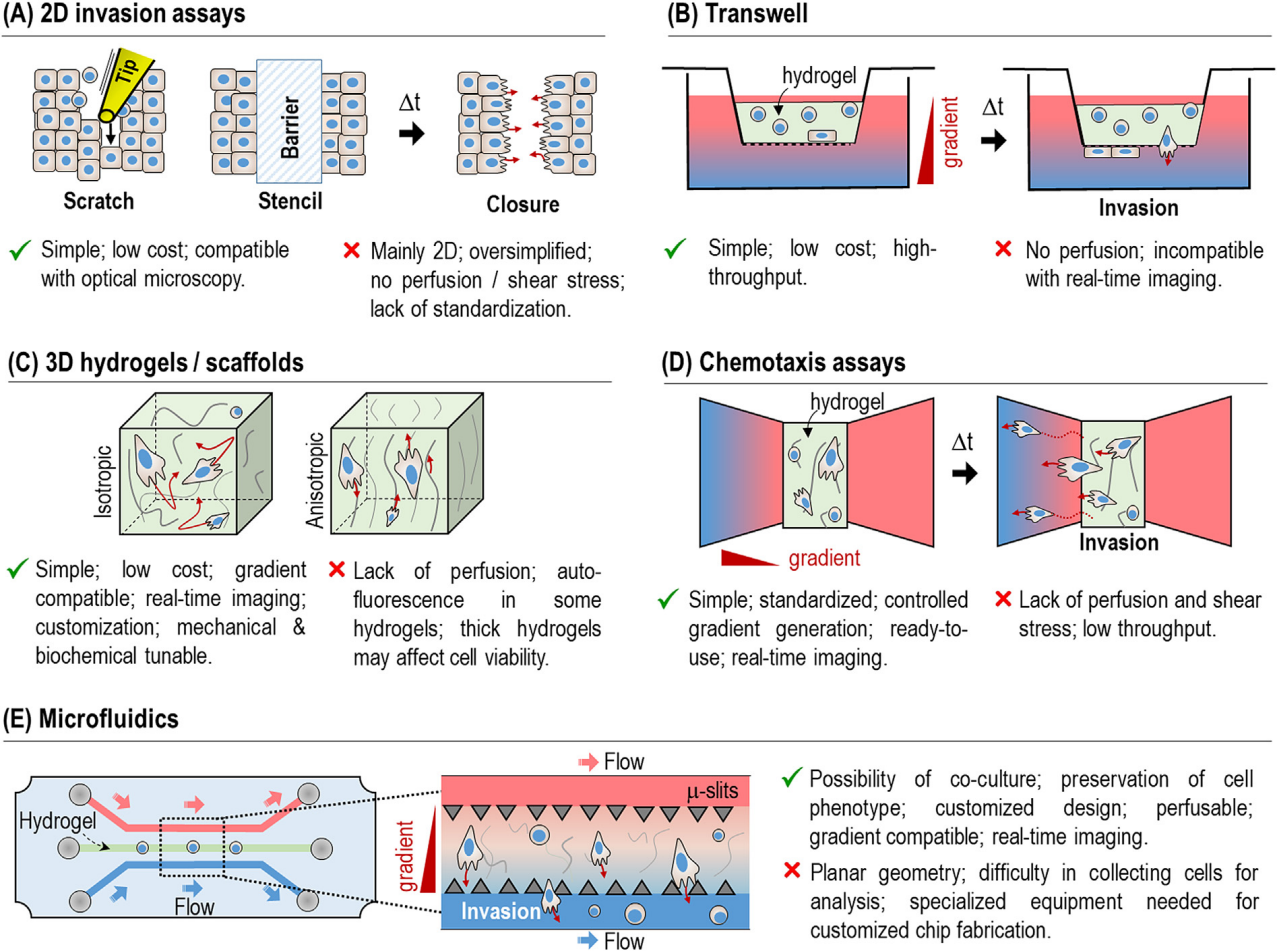
Main methods and assays employed to investigate cell migration. (A) 2D invasion assays, including scratch and stencil-based methods, enable the study of tissue expansion by using two barriers confining the cells. (B) Transwell migration assay with porous membranes with different diameters. (C) 3D hydrogels or scaffolds, with controllable inner architecture (isotropic or anisotropic). (D) Chemotaxis assay for evaluating the chemotactic ability of cells. (E) Microfluidic assay for assessing the effect of fluid flow on the invasion capability of cells.

The selection of the most adequate method depends on the compatibility of the selected approach for measuring specific biophysical parameters (e.g., migration speed, directionality, etc.) or the characterization of cell migration phenotypes (e.g., mesenchymal *vs.* amoeboid). Historically, directed cell migration has been investigated using 2D scratch assays or stencils (Fig. 3A). These are simple, low-cost and well-developed methods to study directed cell migration *in vitro*. The former involves a sterile pipette tip to create a “scratch” in a confluent cell monolayer and monitor the directed motion of the cells closing the generated gap [160,161]. The main drawback of this approach is cells being detached in a non-controlled manner and the uncontrolled damage of the ECM underneath the cells. Stencils can fix this situation by replacing the pipette with microfabricated structures, such as barriers, that restrain cells from migrating. After removing the barrier, they can migrate directionally closing the gap or expanding, depending on the used set-up [162]. Another advantage is the possibility of studying cell expansion by confining cells within a closed region of the stencil. Despite all the advantages of this type of assay, cells migration is limited to a planar environment. Topographically-patterned surfaces enable the cells to migrate in a 3D-like surface while maintaining the simplicity of the assay. Typically, replica molding is employed to 3D pattern the surface of a hydrogel with grooves along which cells can migrate [163,164]. However, cells are not entirely surrounded by an ECM; therefore, they do not mimic their native habitat.

The advent of more realistic 3D cell culture assays has promoted the development of more relevant approaches, including Transwell assays, 3D hydrogels/scaffolds, chemotaxis assays, or microfluidic systems already introduced above (Fig. 3B–E). Transwells are typically employed to evaluate the invasion capabilities of individual cells that migrate through a micro-porous membrane in response to a gradient stimulus (Fig. 3B) [165]. Typically, invasive cells (anchorage-dependent or independent) are seeded in a thin hydrogel layer coating the membrane. After a defined period, the number of cells in the lower chamber is quantified. This method displays several advantages, particularly a high standardization, but in general, it has severe limitations in imaging cell migration. Cells encapsulated within 3D native-like hydrogels (or scaffolds) can overcome this pitfall while providing a native-like habitat (i.e., structural and biochemical) for cells where they display phenotypes and genetic profiles similar to those encountered *in vivo* (Fig. 3C) [166,167]. Typically, collagen or Matrigel are used as a biomimetic matrix due to their superior properties that copycat those from the native scenario, but other hydrogels (or blends) have also been utilized. Importantly, the structural (mechanical and morphological) and biochemical properties of hydrogels can be modulated to mimic those of the cellular microenvironment, such as the fibrillary alignment of the tumour region that facilitate cancer cell invasion. For this, different approaches have been reported, including the stretching of a polymer membrane coated with a hydrogel to align the fibres [168], or the freezing method, which employs two metal plates that directs the linear growth of ice crystals generating aligned fiber bundles [137]. In all these cases, cells migrate directionally by contact guidance but without a preferential direction, i.e., cells can migrate in one or opposite directions. Hydrogels can be combined with 3D gradients of chemokines, even though the slope and extension of the gradient could be very challenging to control to achieve directed migration. Chemotaxis assays can standardize the formation of gradients within hydrogels using microfabricated assays typically containing several interconnected compartments, one to culture the cells (in 2D or 3D) and another used to inject the chemoattractant, promoting the chemotactic migration of the encapsulated cell (Fig. 3D) [169]. There are multiple commercially-available chemotaxis assays, but they can also be manufactured in-house by standard microfabrication techniques [158].

One of the main limitations of this and former methods is the absence of fluid flow, a critical feature involved in cell migration since it provides the needed cues. For this, microfluidics can be employed to investigate many events where (directed) cell migration is applied (Fig. 3E) [[170], [171], [172]]. In typical experimental assays, cells are embedded alone or in co-culture within a hydrogel located inside the microfluidic chip. Usually, adjacent microchannels interconnected to the central one are included to mimic the native vasculature and reproduce the flow of biofluids or the presence of certain chemoattractant stimuli. In specific cases, it is possible to produce the functional unit of a tissue or organ within the microfluidic chip. This approach is typically used to produce on-chip pathophysiological events in an *in vivo*-like manner.

### 3.3 Quantification of cell migration

#### 3.3.1 Imaging methods

Several imaging methods are available to monitor the migration of cells in hydrogels in real-time. Among all the available techniques, standard brightfield, phase contrast, or differential interference microscopy (DIC) stand up due to their simplicity. However, these methods display certain limitations. Brightfield images offer, in general, low contrast, and DIC and phase-contrast imaging add optical artifacts (e.g., bright diffraction halo). In general, it is also difficult to distinguish different cell types without any labelling. More importantly, these techniques are primarily used to image cells that are located on the hydrogel surface due to the difficulty of visualizing their motion when encapsulated within the material, even with careful image processing [173]. Widefield fluorescence and laser-scanning confocal microscopy facilitate cell imaging in 3D hydrogels by collecting fluorescent images in multiple optical planes over time. After acquiring the entire z-stack, the images can be merged to create a detailed 3D movie of cells migrating within the hydrogel. One advantage of confocal microscopy over conventional epifluorescence imaging is the lack of background noise from out-of-focus planes. This is because the physical pinhole filters the interfering light, resulting into higher quality images. Nevertheless, specific deconvolution software/algorithms can be applied to epifluorescence images to improve their quality. This is especially relevant for autofluorescence hydrogels, such as those made of silk fibroin, that may interfere with fluorophores in labelled cells [174]. In this regard, selecting hydrogels with optimal optical properties and good dyes for cell staining is of utmost importance for acquiring high quality images. Typically, cells are labelled with conventional cell membrane inks or transfected (transiently or permanently) with a fluorescent reporter. Cell transfection generally provides better results because the staining does not diffuse over time as for membrane dyes. However, the protocol for generating transfected cells can be technically complex with a moderate efficiency.

A significant limitation of imaging cell migration in 3D hydrogels using fluorescent-based methods is photobleaching and phototoxicity due to the long-term exposition. Therefore, lower acquisition rates are preferred, even though part of the migration path and dynamics of cell are lost to minimize it. More sophisticated optical microscopy techniques have recently emerged to address this problem, particularly light-sheet microscopy. Despite this technique's optical advantages, the manipulation of the sample is still very complex, which limits the type and number of experiments that can be performed [175]. New live cell imaging tools combining high-content screening, robotic manipulation, and automated software analysis/tracking have been developed to improve the amount and quality of data acquired in a faster and more accurate manner [176]. Examples include the FLoid Cell Imaging Station (ThermoFisher Scientific), APX100 (Olympus), Celldiscoverer (Zeiss), or Mica (Leica), among many others Typically, these live cell imaging stations are compatible with 3D multi-color image acquisition, including transmitted light, providing a high-throughput alternative to conventional optical microscopy techniques. More importantly, some of these systems incorporate artificial intelligence for automated sample recognition and data analysis.

#### 3.3.2 Tracking migrating cells

Tracking the motion of individual or collectively migrating cells can provide critical insights regarding their dynamics. Typically, tracking cell trajectories over time is performed from time-lapse movies from which migratory information can be extracted (Table 3). Manual cell tracking remains the gold-standard approach for tracking cells from image sequences, but mainly restricted to cells migrating in 2D surfaces. This method prevents the generation of errors, such as falsely tracked cells, but on the other hand, it is time-consuming, user-dependent, and limits the number of cells that can be sampled. For complex 3D environments, more automated tracking methods have been developed that include the segmentation of the images acquired with fluorescently-labelled cells. (Table 3). Different segmentation methods can be used even though intensity thresholding is the gold standard, allowing the tracking of different sub-groups of cells. This workflow typically generates a data file with quantitative information related to the cell trajectories and dynamics [173].

**Table 3:** An overview of common cell migration tracking tools with their key features and relevant plug-ins.

Tool	Description	Ref.
Image J/Fiji	Typical Plug-in′s include: (i) Manual Tracking and Pointing Cell Tracking: a data set of *x* and *y* coordinates is generated and employed to reconstruct the trajectories of cells, typically 2D. The (semi-) manual tracking mode make the procedure user-dependent and time-consuming. (ii) TrackMate: Segmentation algorithms are employed to detect cell (or organelle) contours and track their trajectories automatically, either in 2D or in 3D. Advanced analytical features provide quantitative data about cell dynamics.	[177] [178] [179]
Cell Tracker	Automatic detection and tracking of cells compatible with both fluorescence and brightfield images. It provides statistical analysis of the cell motion.	[180]
Cell Profiler	Automatic detection and tracking of cells with built-in tools to generate data analysis. Advanced features including machine learning for high-throughput and multi-dimensional image-based data.	[181]
Imaris	Highly sophisticated and accurate algorithms for automatic segmentation, 4D tracking, and analysis of motile objects, such as cells. Quantitative information and statistics about motility analysis is provided.	[182]
LEVER	Collection of software tools for the automatic segmentation, tracking and lineage analysis of individual proliferating cells using phase contrast images. Validation of results and correction of errors can be rapidly performed.	[183]
tTt and qTfy	tTt is a manual single-cell tracking tool which enables the import and interactive inspection of tracking trees exported from other software. qTfy is a supplementary, quantitative tool for multiplexing fluorescence with cell motility attributes.	[184]

Plug-ins for ImageJ or other image processing software can perform automated, semi-automated, and manual cell tracking (Table 3). The performance of each of these methods is highly dependent on the cell density, the complexity of cell displacement during the consecutive frames, or background noise levels. For the latter, some of the available tracking tools include thresholding algorithms to filter out undesired particles (e.g., dust particles) or signals.

#### 3.3.3 Data analysis

**Fig. 4:**
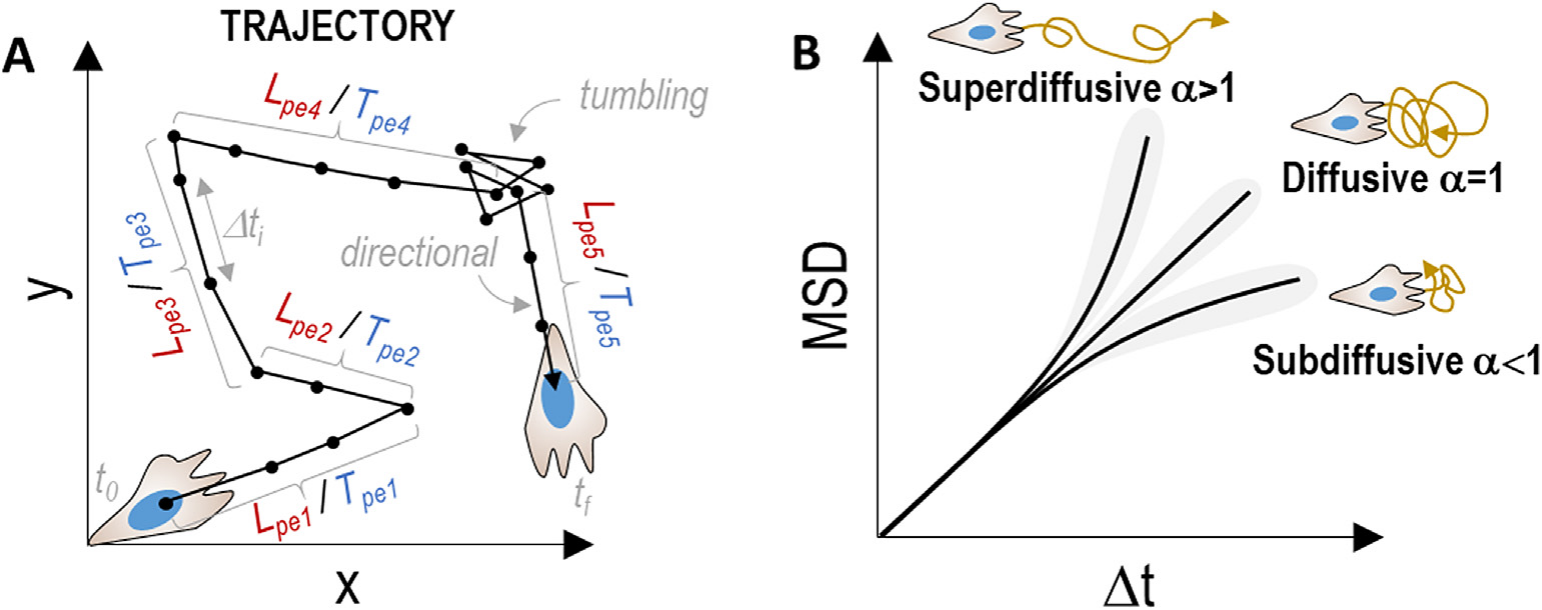
Cell migration analysis. (A) Plot of the trajectory of cells over regular time intervals Δt and representation of Lpe and Tpe parameters to quantify the spatial and temporal persistence of cell migration. For simplicity, this example shows a cell migrating in 2D but the extrapolation to 3D is straightforward. (B) Schematic representation of mean square displacement (MSD) plot for different cell migration modes.

Cell trajectories can be very heterogeneous with cells migrating directionally in response to an external stimulus, moving randomly with no preferential direction, or a combination of both, that is, a sequence of linear movements followed by random trajectories. Some quantitative mathematical parameters have been introduced to quantify the degree of persistence in cell motility [185,186]. Among all of them, cell persistence length/time (Lpe/Tpe) and the mean square displacement (MSD) provide very accurate information about cell invasiveness and dynamics (Fig. 4). Lpe and Tpe are defined as the length and time during which a cell moves directionally without changing direction, respectively. They are measured over the entire cell trajectories and averaged out as shown in Eqs. (1), (2):

(1) $$<Lpe>=\frac{1}{N}\sum_{i=1}^{N}{\left(\frac{1}{n}\sum_{j=1}^{n}{Lpe}_{j}\right)}_{i}$$

(2) $$<Tpe>=\frac{1}{N}\sum_{i=1}^{N}{\left(\frac{1}{n}\sum_{j=1}^{n}{Tpe}_{j}\right)}_{i}$$

where n is the number of linear displacements performed by the cell during the entire trajectories, and N is the total number of cells. Typically, highly invasive cells display elevated Lpe and Tpe values, whereas the cell persistency decreases for randomly migrating cells.

Similarly, the MSD is an excellent quantitative indicator of the degree of directionality of migrating cells over time. It is typically represented by plotting the average displacement of the cell at different time lags. Equation (3) shows the MSD for a single cell migrating in 3D:

(3) $$MSD\left(\tau \right)=\frac{\Delta t}{t_{n-\tau }}\left(\sum_{t=0}^{t_{n}-\tau }\left({\left(x\left(t+\tau \right)-x\left(t\right)\right)}^{2}+{\left(y\left(t+\tau \right)-y\left(t\right)\right)}^{2}+{\left(z\left(t+\tau \right)-z\left(t\right)\right)}^{2}\right)\right)$$

where τ ​= ​nΔt (n ​= ​1, 2 …) and Δt ​= ​time interval between consecutive frames. For multiple cells, the MSD is averaged out as shown in eq. (4):

(4) $$MSD_{all}\left(\tau \right)=\frac{1}{n_{\max}}\sum_{t=0}^{n_{\max}}MSD_{cell\;n}\left(\tau \right)$$

where n_max_ is the total number of timepoints and t_n_ ​= ​nΔt the time lag between the analyzed coordinates.

In general, the MSD is proportional to t^α^, which can be measured from the slope of the MSD *vs* Δt plot (Fig. 4B). A value of α ∼2 indicates a ballistic migration, with cells displaying a highly directional motion, typically responding to a chemoattractant. A value of 2 ​> ​α ​> ​1 indicates a super-diffusive behaviour, that is, cells moving “faster-than-diffusion” and indicating a persistent (spatial and temporal) migration. Next, a value of α ​= ​1 corresponds to diffusive motility (*i.e.,* no directed migration). In this case, the cell displacement is proportional to the time interval. In a *log-log* plot of the MSD, this behavior is represented as a straight line with a slope α ​= ​1. Finally, a value of α ​< ​1 indicates a sub-diffusive nature, where cells move “slower-than-diffusion” indicating a constrained migration. This type of migration behavior is characteristic of cells migrating in crowded – or confined – environments. Finally, the mechanical and biochemical properties of hydrogels (e.g., the spatial distribution of chemokines and adhesion moieties) can influence the value of α and the profile of migrating cells.

However, despite their extensive application MSD and persistent length/time have some limitations, dependent on the acquisition of time lags during imaging. For instance, the uncertainty of MSD coordinates in later timepoints increases when long time intervals span within the trajectory.

## 4 Conclusions

Intense research has been invested in developing biomimetic *in vitro* microenvironments for studying and unravelling the physicochemical mechanisms of cell migration. Among them, hydrogels have become the gold standard materials for engineering 3D matrices recapitulating the properties of the native extracellular milieu. The large diversity and versatility of hydrogels permit the development of realistic environments for monitoring and analysing cell behaviour. In this work, we have given an overview of relevant literature within this field and described and critically reviewed relevant materials, experimental set-ups, and analytical tools to study cell migration in 3D hydrogels. We envision this work as a practical introductory guide for 3D cell migration studies to develop relevant *in vitro* models in biology and disease.

Although recent research in cell migration has advanced rapidly, we are faced with certain challenges that ultimately dictate future prospects for the field. With advances in the areas of artificial intelligence and machine learning, effectively incorporating such technologies into existing analytical platforms for cell migration studies could boost the identification of pathophysiological cell behaviours in a rapid and more automatized manner. Furthermore, the integration of biosensors either within hydrogels or the cell culture platform would provide real-time and localised information related to migrating cells. Together, these would not only yield more physiologically relevant information but also significantly reduce manual input required in order to extract data from migration studies. Next, hydrogels have undoubtedly been an integral part of 3D cell migration studies, with new formulations continually available. Advanced materials that are capable of evolving and are susceptible to changes imposed by migrating cells would provide a more physiologically relevant platform for studying inherent cell migration patterns. Additionally, hydrogels that restructure similarly as native ECM would provide cells with a microenvironment that more accurately replicates the dynamic interdependency between cells and their immediate surroundings. Finally, methods that validate the accuracy of *in vitro* cell migration in hydrogels and their relevance to *in vivo* cell migration are still missing. One approach could be to complement *in vitro* observations with intravital microscopy that enables live cell imaging *in vivo*, providing clarity on the relevance and accuracy of current and future *in vitro* setups. Nevertheless, intravital imaging does not apply to specific body regions, such as the brain. For this reason, developing alternative imaging methods and technologies capable of imaging cell dynamics inside the body in real-time would further provide valuable information about critical pathophysiological phenomena and validate the observations obtained *in vitro* using hydrogels.

## Declaration of competing interest

The authors declare that they have no known competing financial interests or personal relationships that could have appeared to influence the work reported in this paper.

## Data Availability

No data was used for the research described in the article.
